# An efficient algorithm to perform multiple testing in epistasis screening

**DOI:** 10.1186/1471-2105-14-138

**Published:** 2013-04-24

**Authors:** François Van Lishout, Jestinah M Mahachie John, Elena S Gusareva, Victor Urrea, Isabelle Cleynen, Emilie Théâtre, Benoît Charloteaux, Malu Luz Calle, Louis Wehenkel, Kristel Van Steen

**Affiliations:** 1 Systems and Modeling Unit, Montefiore Institute, University of Liège, 4000 Liège, Belgium; 2 Bioinformatics and Modeling, GIGA-R, University of Liège, 4000 Liège, Belgium; 3 Unit of Animal Genomics, GIGA-R and Faculty of Veterinary Medicine, University of Liège, 4000 Liège, Belgium; 4 Unit of Hepato-Gastroenterology, CHU de Liège and Faculty of Medicine, University of Liège, 4000 Liège, Belgium; 5 Department of Gastroenterology, KU Leuven, 3000 Leuven, Belgium; 6 Department of Systems Biology, University of Vic, 08500 Vic, Spain

**Keywords:** Epistasis, Multiple testing, maxT, MB-MDR, GWA studies, Crohn’s disease

## Abstract

**Background:**

Research in epistasis or gene-gene interaction detection for human complex traits has grown over the last few years. It has been marked by promising methodological developments, improved translation efforts of statistical epistasis to biological epistasis and attempts to integrate different omics information sources into the epistasis screening to enhance power. The quest for gene-gene interactions poses severe multiple-testing problems. In this context, the maxT algorithm is one technique to control the false-positive rate. However, the memory needed by this algorithm rises linearly with the amount of hypothesis tests. Gene-gene interaction studies will require a memory proportional to the squared number of SNPs. A genome-wide epistasis search would therefore require terabytes of memory. Hence, cache problems are likely to occur, increasing the computation time. In this work we present a new version of maxT, requiring an amount of memory independent from the number of genetic effects to be investigated. This algorithm was implemented in C++ in our epistasis screening software *MBMDR-3.0.3*. We evaluate the new implementation in terms of memory efficiency and speed using simulated data. The software is illustrated on real-life data for Crohn’s disease.

**Results:**

In the case of a binary (affected/unaffected) trait, the parallel workflow of *MBMDR-3.0.3* analyzes all gene-gene interactions with a dataset of 100,000 SNPs typed on 1000 individuals within 4 days and 9 hours, using 999 permutations of the trait to assess statistical significance, on a cluster composed of 10 blades, containing each four Quad-Core AMD Opteron(tm) Processor 2352 2.1 GHz. In the case of a continuous trait, a similar run takes 9 days. Our program found 14 SNP-SNP interactions with a multiple-testing corrected p-value of less than 0.05 on real-life Crohn’s disease (CD) data.

**Conclusions:**

Our software is the first implementation of the MB-MDR methodology able to solve large-scale SNP-SNP interactions problems within a few days, without using much memory, while adequately controlling the type I error rates. A new implementation to reach genome-wide epistasis screening is under construction. In the context of Crohn’s disease, *MBMDR-3.0.3* could identify epistasis involving regions that are well known in the field and could be explained from a biological point of view. This demonstrates the power of our software to find relevant phenotype-genotype higher-order associations.

## Background

The complete sequence of the human genome has left scientists with rich and extensive information resources. The bloom of bioinformatics, and hence the wide availability of software, has improved the possibility to access and process genomic data. Genome-wide association (GWA) studies, using a dense map of SNPs, have become one of the standard approaches for unraveling the basis of complex genetic diseases [1]. Despite their success, only a modest proportion of currently available heritability estimates can be explained by GWA studies discovered loci [2]. Commonly performed GWA studies usually oversimplify the underlying complex problem, in that usually no account is made for the existence of multiple “small”associations and non-SNP polymorphisms, nor epigenetic effects, genetic pathways, gene-environment and gene-gene interactions [3,4].

A lot of methods and software tools exist to perform large-scale epistasis studies [5]. These Genome-wide Association Interaction (GWAI) studies typically involve balancing between achieving sufficient power, reducing the computational burden and controlling type I error rates. Here, we present a new software tool to perform large-scale epistasis studies, using the MB-MDR methodology [6-9]. MB-MDR is a non-parametric data mining method (no assumptions are made about genetic modes of inheritance) that is able to identify interaction effects for a variety of epistasis models in a powerful way. It is able to distinguish between multiple pure interaction effects and interaction effects induced by important main effects through efficient main effects correction strategies. Apart from identifying multiple sets of significant gene-gene interactions, MB-MDR can also be used to highlight gene-environment interactions in relation to a trait of interest, while efficiently controlling type I error rates. For now, the trait can either be expressed on a binary or continuous scale, or as a censored trait. Extensions to accommodate multivariate outcomes are underway. Here, we mainly focus on second-order gene-gene interactions using bi-allelic genetic markers. However, our software can also handle multi-allelic data and categorical environmental exposure variables, as will be shown in the implementation section. Our C++ software greatly enhances MB-MDR’s first implementation as an R-package [10], both in terms of flexibility and efficiency.

## Implementation

### Input/Output

**Figure 1:**
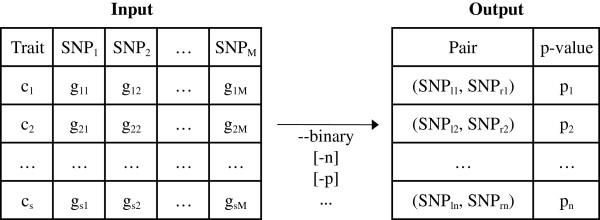
**Input/output formats of *MBMDR-3.0.3. ****MBMDR-3.0.3* takes as argument a text file (possibly converted by our software from PLINK format) containing the trait and SNP values of the subjects under study and a set of command line parameters. If the *a*^*th*^ subject is a case (control), *c*_*a*_=1(0) (*a*=1…*s*). *SNP*_*b*_ is a label referring to the *b*^*th*^ SNP (*b*=1,…*M*). The genotype of an individual *a* at locus *b* is denoted as *g*_*ab*_ (0 if homozygous for the first allele, 1 if heterozygous and 2 if homozygous for the second allele). The produced output is a text file containing the most significant SNP pairs in relation with the trait. (*SNP*_*lj*_,*SNP*_*rj*_) refers to the *j*^*th*^ best SNP pair, i.e. the pair with the *j*^*th*^ lowest p-value *p*_*j*_. Our software has only one mandatory argument: the scale of the trait. Use either −−*binary* for a binary trait, or −−*continuous* for a continuous scale, or −−*survival* for a censored trait (in this case the trait column is replaced by two columns, one for the time variable and one for the censoring variable). We have developed an interactive help, accessible through −−*help*, describing all other options. For instance, *-n* sets the amount of p-values to compute (default: 1000), *-p* sets the amount of permutations to asses statistical significance (default: 999).

The essence of the MB-MDR methodology is to identify sets of gene-gene interactions via a series of association tests, which may or may not be fully non-parametric, while reducing dimensionality. Significance of the explored interactions is assessed using the *maxT* method [11,12] which provides adjusted p-values by controlling for the multiple correlated tests. Then, MB-MDR prioritizes (ranks) the explored interactions via the adjusted p-values. In practical applications, there is an abundance of p-values close or equal to 1 and only a few p-values will point towards interesting multi-locus genotype combination to pursue. With this in mind, we adapt the *maxT* method so that it still calculates the test-statistics for all SNP pairs, but only calculates the p-values of the *n* best pairs, i.e. the ones with the *n* lowest p-values. We show that our method produces the exact same p-values as with the original *maxT* implementation, however using many fewer resources. When interaction signals are expected to be strong in the light of an improved study design (for instance, an increased sample size, a pathway-driven study design, the use of expression traits derived from co-expression networks) or in the context of replicating earlier epistasis findings, the value of *n* should be set sufficiently large by the user, in order not to lose signals in the final output. However, when epistasis is tested for in a hypotheses-free way, it is highly unlikely that more than 1000 significant epistatic pairs will be identified (*n*=1000, default value). Figure 1 gives a description of the input and output formats of the program.

### New implementation of maxT

In this section, we present Van Lishout’s implementation of *maxT* and prove that it requires a memory proportional to *n* (this is: *O*(*n*) memory), whereas the classical implementation of *maxT* requires *O*(*m*) memory. Here, *m* and *n* refer to the number of SNP pairs and the number of top pairs to retain in the output, respectively.

**Figure 2:**
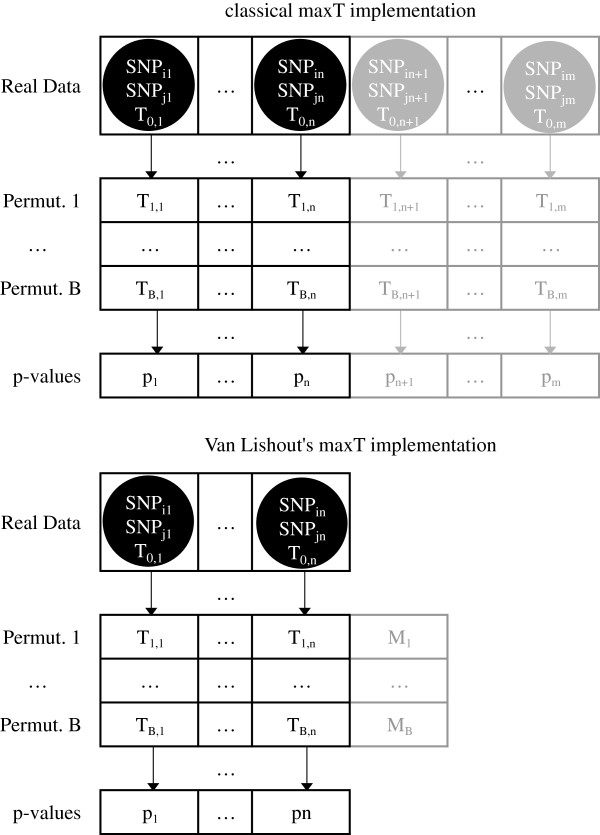
**Classical versus Van Lishout’s implementation of maxT.** In the classical *maxT* implementation, all *T*_*i*,*j*_ values are in memory. If only the *x* best p-values are envisaged then only the maximum *M*_1_,…,*M*_*B*_ of the [*T*_1,*n*+1_,…,*T*_1,*m*_],…,[*T*_*B*,*n*+1_,…,*T*_*B*,*m*_] are needed, implying only temporary storage of the corresponding values.

The different steps of the original *maxT* algorithm can be decomposed as follows (see [11] for a detailed explanation of the logic behind these steps): 

1. Compute the test-statistics for all pairs of SNPs (*j*=1,…,*m*) and sort them. The result is the *Real Data* vector of Figure 2 where *T*_0,1_≥*T*_0,2_≥…≥*T*_0,*m*_.

2. Generate *B* random permutations of the trait column. For each permutation *i*=1,…,*B*, compute the test-statistics *T*_*i*,*j*_ for all pairs of SNPs (*j*=1,…,*m*) in the order defined by the *Real Data* vector. Force the monotonicity of the rows: for *j*=*m*−1,…,1 replace *T*_*i*,*j*_ by *T*_*i*,*j*+1_ if *T*_*i*,*j*_<*T*_*i*,*j*+1_.

3. For each pair of SNPs *j*=1,…,*m* compute the number *a*_*j*_ of *T*_*i*,*j*_ values such that *T*_*i*,*j*_≥*T*_0,*j*_, for *i*=0,…,*B*.

4. Compute the p-values using the equation $p_{j}=\frac{a_{j}}{B+1}$ for each pair of SNPs. Force the monotonicity of the p-values: for *j*=1,…,*m*−1 replace *p*_*j*+1_ by *p*_*j*_ if *p*_*j*+1_<*p*_*j*_.

Note that the intuition behind the monotonicity enforcing procedure at step 2 is to correct the test-statistics that are obviously too pessimistic: the test-statistic of a pair *P*1 should not be lower than the test-statistic of a less significant pair *P*2. Replacing the test-statistic computed for *P*_1_ by the one computed for *P*_2_ is therefore a better estimation of the significance of *P*_1_. The amount of false-negative results would be higher without this procedure. Similarily, the purpose of the monotonicity enforcing procedure at step 4 is to correct p-values that are obviously too optimistic: the p-value of *P*_2_ should not be lower than the p-value of *P*_1_. Replacing the p-value computed for *P*_2_ by the one computed for *P*_1_ is therefore a better estimation of the significance of *P*_2_. The amount of false-positive results would be higher without this final step.

From a memory point of view, it is best to implement the aforementioned algorithm in a slightly different way. Indeed, the current description implies all *Permutation* vectors of Figure 2 to be in memory at the same time. This requires *O*(*Bm*) memory. In fact, a memory of *O*(*m*) can be achieved by adopting a different approach. The idea is that the *a*_*j*_ values calculated at step 3, can already be calculated on-the-fly. A vector *a-values* of all *a*_*j*_ values can be created from scratch and initialized with 1’s values. Indeed, note that at step 3 the original sample series counts as 1 of B+1 available samples to assess significance. For *i*=0,*T*_0,*j*_≥*T*_0,*j*_ and hence *a*_*j*_=1,∀*j*=1,…,*m*. The elements of the *a-values* vector can then be updated at the end of each iteration *i*=1,…,*B* of step 2 by incrementing the *a*_*j*_ values corresponding to the *T*_*i*,*j*_≥*T*_0,*j*_ by one. In this way, all *i*^*th*^*T*_*i*,*j*_ values can be removed from memory at the end of the *i*^*th*^ iteration since they are no longer of any use. Hence, only a single *Permutation* vector is stored instead of *B* vectors. In fact, applying step 4 to the *a-values* vector obtained at the end of this procedure readily leads to the final *p-values* vector.

This proves that this algorithm requires *O*(*m*) memory. Obviously, if *M* denotes the number of SNPs, *m* is given by the formula *m*=*M*(*M*−1)/2. The memory usage of the classical implementation thus rises quadratically with the number of SNPs, whereas we will now see that our method is independent of it.

The monotonicity enforcing process executed at the end of step 2, implies that we need to calculate all *T*_*i*,*j*_ values, even if we are only interested in the first *n* p-values. However, not all of these *T*_*i*,*j*_ values have to be stored in memory. For our purpose, only *T*_*i*,*j*_(1≤*j*≤*n*) and *M*_*i*_, the maximum of the [*T*_*i*,*n*+1_,…,*T*_*i*,*m*_] elements, are required. In other words, there is no need to explicitly propagate *M*_*i*_ to position *n*+1. It suffices to compute *M*_*i*_ and to replace *T*_*i*,*n*_ by *M*_*i*_ if and only if *M*_*i*_>*T*_*i*,*n*_. The monotonicity enforcement continues from positions *n*−1 through 1.

The different steps of our algorithm, exploiting all ideas presented so far, are given below: 

1. Compute the test-statistics for all pairs but store only the *n* highest ones. The result is a *Real data* vector where *T*_0,1_≥*T*_0,2_≥…≥*T*_0,*n*_.

2. Initialize a vector *a* of size *n* with 1’s.

3. Perform the following operations for *i*=1,…,*B*: 

(a) Generate a random permutation of the trait column.

(a) Compute the test-statistics *T*_*i*,1_,…,*T*_*i*,*n*_ and store them in a *Permutation*_*i*_ vector.

(a) Compute the maximum *M*_*i*_ of the test-statistics values *T*_*i*,*n*+1_,…,*T*_*i*,*m*_.

(a) Replace *T*_*i*,*n*_ by *M*_*i*_ if *T*_*i*,*n*_<*M*_*i*_.

(a) Force the monotonicity of the *Permutation*_*i*_ vector: for *j*=*n*−1,…,1 replace *T*_*i*,*j*_ by *T*_*i*,*j*+1_ if *T*_*i*,*j*_<*T*_*i*,*j*+1_.

(a) For each *j*=1,…,*n*, if *T*_*i*,*j*_≥*T*_0,*j*_ increment *a*_*j*_ by one.

4. Divide all values of vector *a* by *B*+1 to obtain the *p-values* vector *p*. Force monotonicity as follows: for *j*=1,…,*n*−1, replace *p*_*j*+1_ by *p*_*j*_ if *p*_*j*+1_<*p*_*j*_.

Two remarks are in place:

First, the main idea of the *Sorting by insertion* algorithm [13] can be recycled to perform step 1 using *O*(*n*) memory. The *Real Data* vector is first initialized with the first *n* computed test-statistics and sorted using the quick sort algorithm [13]. Then, at each iteration, the next test-statistic is calculated and compared with the smallest value of the vector. If it is smaller or equal nothing has to be done. Otherwise, the smallest value is removed and the new one is inserted in order to preserve the sorting. This insertion requires $\frac{n}{2}$ operations on average. This method works particularly well on large-scale problems, where *m*>>*n*. Intuitively, the probability of having to insert will decrease at each iteration and tend to zero because the *Real Data* vector will contain higher and higher values. This algorithm will take *O*(*m*) time on average, but could degenerate in *O*(*nm*), which is still linear.

Second, it should be noted that step 3(b) and 3(c) can be merged into a single step. The idea is to create first a hash table containing the indexes of the *n* best pairs, resolving collision by separate chaining [13]. The test-statistics *T*_*i*,*j*_ can then be computed in any convenient order. At each iteration, the hash table is used to decide (almost instantaneously) if the current value corresponds to one of the *n* best pairs or not, and perform either step 3(b) or step 3(c) accordingly.

### Parallel workflow

**Figure 3:**
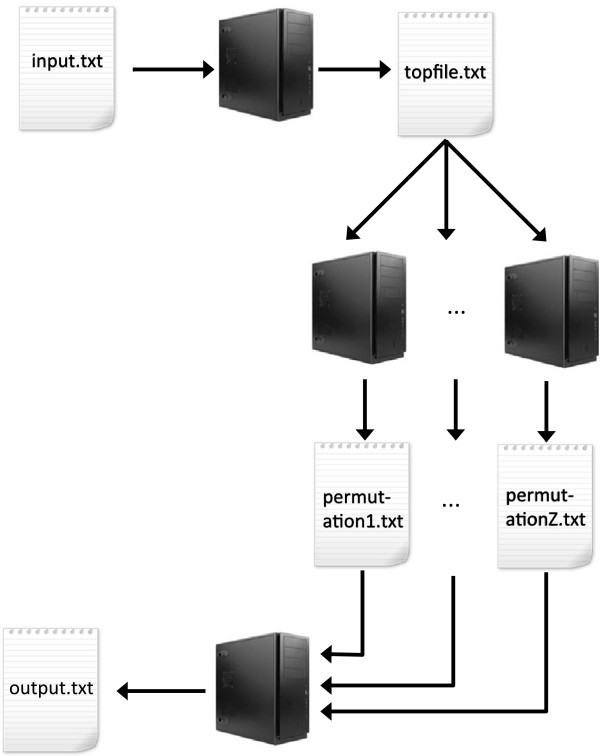
***MBMDR-3.0.3 *parallel workflow.** Step 1 of the maxT algorithm is first performed on the input file. This produce the file topfile.txt, containing the top pairs of SNPs and their corresponding test-statistics. Then, the computation of the permutations is split between the available machines. Finally, MBMDR-3.0.3 reads the produced permutation_*x*_*.txt* files to create the final output file.

Since the memory used by Van Lishout’s implementation of *maxT* is independent from the number of SNPs, memory is no longer a problem. However, the remaining concern is time. Since all iterations of the 3rd step in the new *maxT* implementation are independent from each other, it is possible to simultaneously run the computations of the permutations on different machines. Figure 3 describes the three steps of the parallel workflow that we use to solve large-scale problems: 

1. Compute the test-statistics for all pairs on one machine and save the *n* highest ones into a file *topfile.txt*. This file should be saved at a location on which each machine has read access. It will contain the information of the *Real Data* vector of Figure 2 and have thus a size of only *O*(*n*).

2. Split the computation of the permutations homogeneously between the Z machines. On each machine z=1…Z, perform the following operations: 

(a) Read the file topfile.txt

(a) Initialize a vector p of size n with 0’s.

(a) Execute step 3 of Van Lishout’s maxT algorithm for each permutation assigned to z (using vector p instead of a).

(a) Save the p vector into a file *permutation*_*z*_*.txt.*

3. When all machines have terminated their work, sum all vectors of the files *permutation*_*1*_*.txt…permutation*_*Z*_*.txt* to obtain a vector p. Perform step 4 of Van Lishout’s maxT algorithm on this vector.

However, the main feature that makes our software fast is not parallelization, but speed of the test-statistic computations. Indeed, we have seen that the *maxT* algorithm computes *B×mT*_*i,j*_ values. Solving *B*=999 permutations with a dataset of *M*=100,000 SNPs, i.e. *m=O*(10^*10*^) pairs of SNPs, means thus *O*(10^*13*^) computations to perform. It is obvious that the computation of the test-statistic *T*_*i,j*_ has to be very fast and that each improvement can have a dramatic influence on the final computing time. We show in the next section how we achieve this goal.

### Test-statistic computation

This section presents the implementation of the computation of the *T*_*i,j*_ values, capturing the degree of association between the *j*^*th*^ pair of SNPs [*SNP*_*lj*_*,SNP*_*rj*_] and the *i*^*th*^ permutation of the trait *Trait*_*i*_. Let M+1 (n+1) be the number of possible values for *SNP*_*lj*_ (*SNP*_*rj*_). In practice, most of the studies concern bi-allelic genetic markers and M=N=2. However, our program automatically detects the exact values of M and N, so that multi-allelic variables are also covered. Furthermore, categorical environment variables can also be handled, as long as they are coded 0, 1,... M (N).

Since we are interested in solving large-scale problems, we must realize that the part of the code that reads the dataset at the start of the program cannot store it in cache because of its size. Accessing to the trait and SNP values is thus slow and must be avoided as much as possible. For this reason, the three columns of interest (*Trait*_*i*_, *SNP*_*lj*_ and *SNP*_*rj*_) will be passed by value and not by reference to the function. In this way, an explicit local copy of them will be performed, on which the function will be able to work faster.

**Figure 4:**
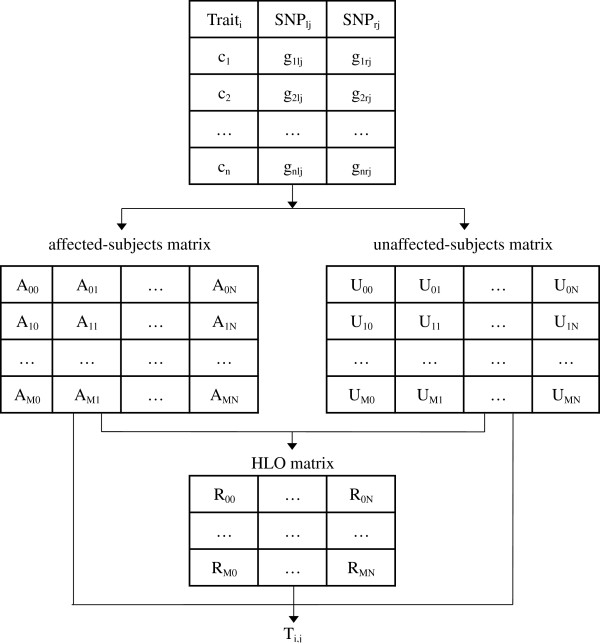
Decomposition of the different steps of the computation of *T*_*i,j*_. *c*_*a*_ is 1 (0) if the *a*^*th*^ subject is a case (control) for the *i*^*th*^ permutation of the trait. *g*_*alj*_ and *g*_*arj*_ are 0, 1 or 2 depending on the genotype of the *a*^*th*^ subject for the *j*^*th*^ pair. *A*_*mn*_ and *U*_*mn*_ are respectively the number of affected/unaffected subjects, whose genotype *g*_*kl*_= *m* and *g*_*kr*_= *n. R*_*mn*_ is either “H” if the subjects whose genotype is *m* for *SNP*_*lj*_ and *n* for *SNP*_*rj*_ have a high statistical risk of disease, “L” if they have a low statistical risk and “O” if there is no statistical evidence.

Different options are implemented at the different steps of the computation of *T*_*i,j*_, depending on the nature of the trait (e.g. [8,14,15]). Figure 4 illustrates the three main steps involved in the statistics computations, in the case of a case-control or cohort design and a binary (affected/unaffected) trait, without adjusting for main effect, speeding up the computation time. Similar mechanisms hold for other MB-MDR eligible scenario’s. 

1. Generation of the affected-subjects and unaffected-subjects matrices. These matrices are easily obtained by performing a loop over the subjects of the dataset: for a=1,…n, if *c*_*a*_=1 increment a cell of the affected-subjects matrix, else a cell of the unaffected-subjects matrix. The cell to be incremented depends on the genotype: *g*_*alj*_ indicates which row of the matrix has to be incremented and *g*_*arj*_ which column.

2. Generation of the HLO-matrix from the two matrices generated at step 1. The value of each *R*_*mn*_ elements depends on a test for association between the trait and the genotype (*SNP*_*lj*_ = *m*, *SNP*_*rj*_ = *n*). This can be a *χ*^*2*^ test with one degree of freedom in the case of a binary trait, an F-test in the case of a continuous trait, a log-rank test in the case of survival data. However, the architecture of the software makes it easy to implement other test statistics that are appropriate for the data at hand. For binary traits, the implemented test statistic is defined by$\frac{{\left(\text{ad}-\text{bc}\right)}^{2}(a+b+c+d)}{\left(a+b)(c+d)(b+d)(a+c\right)}$, where a and b refer to the number of affected and unaffected subjects having the genotype (*SNP*_*lj*_ = *m*, *SNP*_*rj*_ = *n*) and c and d refer to the number of affected and unaffected subjects having a different genotype. This statistic follows a *χ*^*2*^ distribution. If we define *N*_*A*_ and *N*_*U*_ to be the total number of affected and unaffected subjects, those values are easy to compute: *a = A*_*mn*_,*b = U*_*mn*_,*c = N*_*A*_*−A*_*mn*_ and *d = N*_*U*_*−U*_*mn*_. At this point, if either a+b or c+d is below a threshold that is a parameter of the program (default value 10) then the test is not performed at all, since it would not be statistically significant. In this case the value of *R*_*mn*_ will be set to “O”, to indicate the absence of evidence that the subset of individuals with multilocus genotype (*SNP*_*lj*_ = *m, SNP*_*rj*_ = n) has neither a high nor a low risk for disease. Otherwise, the test is performed. When the computed *χ*^*2*^ value is not significant based on a liberal significance threshold of 0.1 (default value in the software), the value of *R*_*mn*_ will be set to “O”, to indicate that we cannot reject the independence hypothesis. Otherwise, *R*_*mn*_ will be set to either “H” if (ad−bc)>0, to indicate that the population whose genotype is (*SNP*_*lj*_ = *m, SNP*_*rj*_ = *n*) has a high risk of having the trait, or to “L” if (ad−bc)<0, to indicate a low risk for this event.

3. Computation of *T*_*i,j*_ from the three matrices generated at step 1 and 2. It consists in performing two *χ*^*2*^ tests with one degree of freedom and returning the maximum of both. The first one tests association between the trait and the belonging to the “H” category versus the “L” or “O” category. The second one tests association between the trait and the belonging to the “L” category versus the “H” or “O” category. In the first (second) case, a and b are respectively the number of affected and unaffected subjects belonging to the “H” (“L”) category and c and d to the “L” (“H”) or “O” category. Computing this can be easily achieved by initializing a,b,c and d to zero, and for each *R*_*mn*_ adding *A*_*mn*_ to a and *U*_*mn*_ to b if *R*_*mn*_ = “H” (“L”) and *A*_*mn*_ to *c* and *U*_*mn*_ to d otherwise.

In summary, this paragraph shows that to make this methodology fast, reading the data of the subjects only once during step 1 to create the affected-subjects and unaffected-subjects matrices is a key. In this way, the test statistic computation function can quickly start to work on a very small part of memory that is in cache. The keys that make step 2 and 3 fast are respectively the fact that computing an *R*_*mn*_ value does not require any loop and the fact that a single loop of nine iterations (in the bi-allelic case) allows to calculate all the numbers needed in the *χ*^*2*^ formula.

## Results and discussion

Here we present results for both simulated data and real-life data.

### Simulated data

In order to assess the speed performances of our C++ software MBMDR-3.0.3, we created 4 different datasets with 1,000 individuals each, of respectively 100 SNPs, 1,000 SNPS, 10,000 SNPs and 100,000 SNPs. To assess significance of MB-MDR results, the number of permutations was set to B=999. Each dataset was constructed to contain a strong signal for the functional pair [*SNP*_*5*_,*SNP*_*10*_]. Table 1 states the two-locus penetrance table used to generate the data. A balanced number of cases and controls is sampled. The minor allele frequencies of the functional SNPs were fixed at 0.5 and those of the non-functional SNPs were generated randomly from a uniform distribution on [0.05, 0.5]. This corresponds to the first of six purely epistatic models discussed in [16]. A similar strategy was used to construct another set of 4 datasets, containing the same number of individuals and SNPs as before, but expressing the trait on a continuous scale instead of a binary one. MBMDR-3.0.3 finds the strong signal in all datasets. Table 2 gives the execution times. Since the parallel workflow of MBMDR-3.0.3 was tested on a cluster composed of 10 blades, containing each four Quad-Core AMD Opteron(tm) Processor 2352 2.1 GHz, the computation of the permutations was split between 10×4×4=160 cores for this experiment. Table 2 shows that our software is about two times faster for solving datasets for which the trait is expressed on a binary scale, compared to datasets where the trait is expressed on a continuous one. Finally, the results in Table 2 also show that the execution time is approximately multiplied by 100 when the number of SNPs is multiplied by 10. This is logical, since the computation time mainly depends on how many test-statistics are computed, which in turn depends on the quantity of pairs of SNPs, which is proportional to the squared number of SNPs.

**Table 1:** Two-locus penetrance table used to create the strong signal

	**b/b**	**b/B**	**B/B**
a/a	0	0.1	0
a/A	0.1	0	0.1
A/A	0	0.1	0

**Table 2:** **Execution times of *MBMDR-3.0.3***

****SNPs****	***MBMDR-3.0.3***	***MBMDR-3.0.3***	***MBMDR-3.0.3***	***MBMDR-3.0.3***
	**sequential execution**	**sequential execution**	**parallel workflow**	**parallel workflow**
	**Binary trait**	**Continuous trait**	**Binary trait**	**Continuous trait**
100	45 sec	1 min 35 sec	<1sec	<1sec
1,000	1 hour 16 minutes	2 hours 39 minutes	38 sec	1 min 17 sec
10,000	5 days 13 hours	11 days 19 hours	1 hour 3 min	2 hours 14 min
100,000	≈ 1.5 year	≈ 3 years	4 days 9 hours	≈ 9 days

The parallel workflow was tested on a cluster composed of 10 blades, containing each four Quad-Core AMD Opteron(tm) Processor 2352 2.1 GHz. The sequential executions were performed on a single core of this cluster. The results prefixed by the symbol “ ≈” are extrapolated.

### Crohn’s disease data

We apply our software to real-life data on Crohn’s disease [17][18]. Here, Caucasian Crohn’s disease patients and healthy controls are genotyped using Illumina HumanHap. Quality control tests are performed on these data excluding SNPs and individuals with more than 5% missing genotypes. Individuals with mean heterozygosity outside the range of 31% to 38% are discarded. The gender of the individuals is predicted from the mean homozygosity on X markers and samples with contradiction between the estimated and the recorded gender are excluded. SNPs violating Hardy-Weinberg principle are discarded using a*χ* p-value threshold of 10^−4^. Related individuals are identified using pairwise IBS tests and discarded as well. The cleansing process give rise to a set of 1687 unrelated Caucasians (676 CD patients and 1011 healthy controls) and 311,192 SNPs.

For the purpose of this study, we use Biofilter.0.5.1 [19] as an additional data preparation step. It uses a knowledge-driven approach to prioritize genetic markers in gene-gene interaction screening while reducing the search space. In particular, Biofilter allows the explicit detection and modeling of interactions between a large set of SNPs based on biological information about gene-gene relationships and gene-disease relationships. The knowledge-based support for the models is attributed by implication index, which is simply a number of data sources that provide evidence of gene-gene interaction or gene-disease relationship, and is calculated by summing the number of data sources supporting each of the two genes and the connection between them (see [19] for more details). In practice, to make the prioritization procedure in Biofilter more focused on CD, we apply a list of candidate genes for CD (120 genes collected from the publications [18][20][23]) and 160 groups (collected basing on selective search in Biofilter using keywords*crohn, enteritis, inflam, autoimmune, immune, bowel, gastrointest, ileum, ileitis, intestine, lleocolic, diarrhea, stenosis* and *cytokine*). Using this approach/analysis we ended up with 12,471 SNPs that we further analyze in MB-MDR.

**Figure 5:**
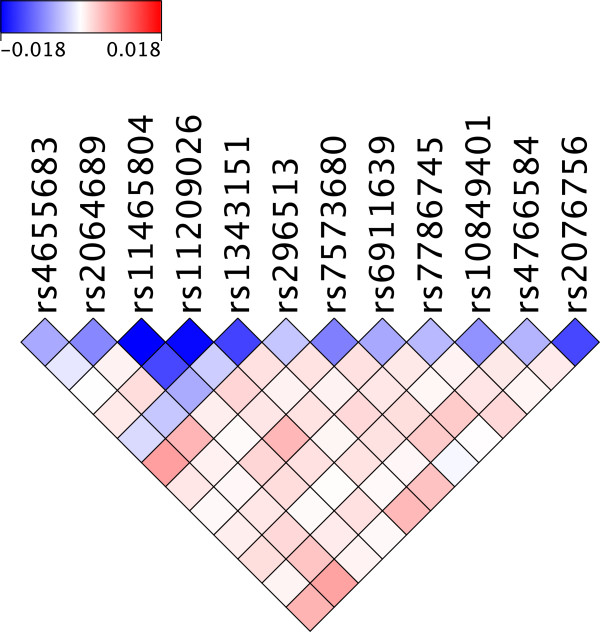
**SD plot.** Synergy Disequilibrium (SD) plot of potential epistasis interactions between the loci indicated in Table 3

Table 3 lists all the *MBMDR-3.0.3* statistically significant interactions for the Crohn’s disease data under investigation. Note that these results are adjusted for testing about 77 million pairs of SNPs. Table 4 shows the genomic location of SNPs involved in these interactions. A total of 13 out of 14 significant interactions involves rs11209026 or rs11465804. Both SNPs are located in the interleukin-23 receptor (*IL23R*) gene, a known susceptibility gene for CD. The SNP rs11209026 is a non-synonymous coding SNP (Arg381Gln substitution), while rs11465804 is intronic and in strong linkage disequilibrium (LD) with rs11209026 (*r*^**2**^ = 0.97). In the original GWA studies, these two SNPs gave the most significant association signals with *p* < 10 ^−9^. Given the strong correlation between the SNPs, it is to be expected that all interactions found for one SNP are also found for the other (Table 3). The most significant interaction is between rs11209026 and rs7573680 (p=0.004). The latter is an intronic SNP located in *HDAC4* (histone deacetylase 4). Figure 5 shows a synergy disequilibrium plot [24] for the SNPs listed in Table 3. Such a plot is able to highlight disease-associated haplotypes, as well as epistatically interacting loci with respect to disease. Interestingly, when we adjust the MB-MDR screen for main effects [9], no significant SNP pair is relevant.

**Table 3:** SNP-SNP interactions having a multiple testing corrected p-value < 0.05

**First SNP**	**Second SNP**	**p-value**
rs11209026	rs7573680	0.004
rs11465804	rs7573680	0.017
rs11209026	rs2064689	0.018
rs11209026	rs6911639	0.021
rs11209026	rs4766584	0.023
rs11465804	rs2064689	0.025
rs11465804	rs4766584	0.028
rs11465804	rs6911639	0.029
rs11465804	rs10849401	0.033
rs11209026	rs296513	0.037
rs1343151	rs2076756	0.04
rs11209026	rs10849401	0.044
rs11209026	rs7786745	0.048
rs11209026	rs4655683	0.048

**Table 4:** Location of the SNPs involved in a significant SNP-SNP interaction

**SNP**	**Position**	**Gene**
rs11209026	chr1:67705958	**IL23R**
rs11465804	chr1:67702526	*IL23R*
rs7573680	chr2:240169077	*HDAC4*
rs2064689	chr1:67653010	*IL23R*
rs6911639	chr6:32978178	*HLA-DOA*
rs4766584	chr12:109663581	*ACACB*
rs296513	chr1:200906473	*C1orf81*
rs10849401	chr12:6273238	intergenic
rs7786745	chr7:18422684	intergenic
rs4655683	chr1:67611613	intergenic
rs1343151	chr1:67719129	*IL23R*
rs2076756	chr16:50756881	*NOD2*

Locations obtained by GenomeBuild 37.1.

### Discussion

Several studies have suggested that different signals exist in *IL23R*, conferring risk or protection to Crohn’s disease. A study by Taylor *et al*[25], where they aimed to estimate the total contribution of the *IL23R* gene to CD risk using a haplotype approach, showed that the population attributable risk for these haplotypes was substantially larger than that estimated for the *IL23R* Arg381Gln variant alone. *MBMDR-3.0.3* identified several “epistatic” signals from pairs of SNPs located in the *IL23R* gene. It should be noted though that epistasis signals on SNPs in LD are considered to be non-synergetic. The MB-MDR discoveries on Crohn’s disease also seem to give us a new working hypothesis to expand on the current knowledge (histone deacetylation). Indeed, histone deacetylation results in a compact chromatin structure commonly associated with repressed gene transcription (epigenetic repression), and hereby plays a critical role in transcriptional regulation, cell cycle progression and developmental events. Although not known to physically interact directly, IL23R and HDAC4 could be linked trough MAPK1/STAT3 signaling: MAPK1 has been shown to associate with phosphorylate HDAC4 [26]. Protein phosphorylation regulates the corepressor activity of the deacetylase. MAPK1 also acts as an important activator of STAT3 (signal transducer and activator of transcription 3) which is an essential regulator of immune-mediated inflammation. In addition, the IL23/IL23R pathway modulates STAT3 transcriptional activity, and recently it has been shown that CD8+ T cells from Arg381Gln *IL23R* carriers showed decreased STAT3 activation compared with WT CD8+ T cells [27]. It can thus be hypothesized that a balanced action between the HDAC1/MAPK1 and IL23/IL23R pathways, converging on STAT3 signaling, are important for CD pathogenesis. The fact that no significant SNP pairs remain, following an adjustment of the MB-MDR screen for main effects (an observation that already emerged after interpreting Figure 5) seems to suggest that the significant results for the SNP pairs of Table 3 are mainly induced by important main effect players.

*MBMDR-3.0.3* can accommodate a variety of study designs and outcome types, can correct for important lower order effects and satisfactory deals with the computational burden induced by highly-dimensional complex data. In order to upscale the applicability of the MB-MDR methodology towards genome-wide association interaction analyzes, the method was implemented in C++ and a new version of the *maxT* algorithm was incorporated. This version requires an amount of memory that is independent from the number of genetic effects to be investigated. We were able to further reduce the execution time, first by parallelizing the processes and second by optimizing the test-statistic function capturing the degree of association between a pair of SNPs and a trait. All of these features, available in *MBMDR-3.0.3*, are promising in the light of GWAI studies. Alternative approaches to deal with execution time are proposed, for example GPU [28] and cloud computing [29]. Used in conjunction with MB-MDR, those methods could lead to very fast software tool to solve GWAI studies problems.

## Conclusions

In this paper we have presented the epistasis screening software *MBMDR-3.0.3*. It is based on a new implementation of *maxT*. The main advantage of this improvement, is that it solves memory problems for any kind of analysis by becoming independent from the number of SNPs, without loss of power. We have also presented a fast implementation of a test-statistic function indicating the association between the trait and a pair of SNPs.

We have tested our program on simulated datasets of increasing size. The parallel workflow was tested on a cluster composed of 10 blades, containing each four Quad-Core AMD Opteron(tm) Processor 2352 2.1 GHz and is able to analyze all pairwise gene-gene interactions with a dataset of 100,000 SNPs typed on 1000 individuals within 4 days and 9 hours, using 999 permutations of the trait to assess statistical significance.

## Availability and requirements

•**Project name:** MB-MDR

•**Project home page:**http://www.statgen.ulg.ac.be

•**Operating system(s):** Mac OS X and Linux

•**Programming language:** C++

•**Restrictions on use by non-academics:** no limitations

## Competing interests

The authors declare that they have no competing interests.

## Authors’ contributions

FVL had the original idea of the new maxT algorithm and LW helped actively on its formalization. FVL is the developer of all versions of our C++ software, from *MBMDR-1.0.0* to *MBMDR-3.0.3* and the principal author of the manuscript. VU and JM took an active role in the analysis part of the software development. JM and EG tested the program intensively on both simulated and real-life datasets. ET and BC performed the quality control of the Crohn’s disease real-life dataset and EG used Biofilter to select a subset of interesting SNPs from it. IC is the main contributor of the discussion paragraph on the Crohn’s disease results. KVS, MC and VU were engaged in all stages of the project. All authors read and approved the final manuscript.
